# NPM1-rearranged AML: clinical features, molecular pathogenesis, and therapeutic perspectives

**DOI:** 10.1007/s12185-026-04234-x

**Published:** 2026-06-05

**Authors:** Yuko Shimosato, Susumu Goyama

**Affiliations:** Division of Molecular Oncology, Department of Computational Biology and Medical Sciences, Graduate School of Frontier Sciences, The University of Tokyo, 4-6-1 Shirokanedai, Minato-ku, Tokyo 108-8639 Japan

**Keywords:** NPM1 rearrangements, NPM1c, XPO1, Menin, HOX genes

## Abstract

Nucleophosmin 1 (*NPM1*) gene aberrations are among the most common genetic alterations in acute myeloid leukemia (AML). *NPM1* mutations (NPM1c), detected in 30–40% of adult AML cases, are well-characterized and associated with distinct clinical and molecular features. In contrast, *NPM1* rearrangements (NPM1-r), involving rare fusion partners such as *MLF1, CCDC28A, HAUS1*, and *RARA,* are much less common and have historically been poorly understood. However, recent studies have begun to shed light on the molecular mechanisms and clinical characteristics of NPM1-r AML, revealing overlapping features with NPM1c-AML. Both types share key leukemogenic mechanisms, including cytoplasmic mislocalization of NPM1, interaction with XPO1, and sustained *HOX* gene expression, which drive leukemic transformation. NPM1-r AML may exhibit unique clinical characteristics, including a higher prevalence in younger patients and potentially a poorer prognosis, although further validation in larger cohorts is needed. Therapeutic strategies targeting XPO1 and Menin, which have shown promise in NPM1c-AML, may also hold potential for NPM1-r AML. Continued research is essential to further elucidate the biology of this rare AML subtype and to establish optimized treatment strategies.

## Introduction

Aberrations in the nucleophosmin (*NPM1*) gene represent one of the most common genomic lesions in acute myeloid leukemia (AML). *NPM1* mutations are detected in approximately 5% of pediatric AML cases and in 30–40% of adult AML cases, with the frequency rising to 40–50% in adult AML with a normal karyotype [1]. These mutations are almost invariably heterozygous, with the majority consisting of a four-base pair insertion in exon 12. This insertion causes a frameshift that generates a novel nuclear export sequence. While wild-type NPM1 predominantly localizes to the nucleolus, mutant NPM1 proteins are redistributed to the cytoplasm and are therefore referred to as cytoplasmic NPM1 (NPM1c) [2].

NPM1c frequently co-occurs with mutations in *FLT3* (particularly *FLT3-ITD*), *DNMT3A*, *IDH1*, and *IDH2*, suggesting cooperative interactions between NPM1c and these mutations in driving leukemogenesis. Conversely, *NPM1* mutations are largely mutually exclusive with mutations in *RUNX1*, *CEBPA*, and recurrent fusion oncogenes such as *RUNX1-ETO, CBFB-MYH11*, and *MLL* rearrangements [3]. Clinically, prior to the widespread use of FLT3 inhibitors, NPM1c-AML was associated with a relatively favorable prognosis in the absence of *FLT3-ITD* or when *FLT3*-ITD was present at a low allelic ratio. However, cases with a high *FLT3*-ITD allelic ratio were classified as intermediate risk. Furthermore, NPM1c-AML harboring mutations in *WT1* or co-mutations involving *FLT3*-ITD and *DNMT3A* is known to confer an adverse prognosis [4].

In addition to the commonly detected frameshift mutations in exon 12, studies have identified balanced chromosome rearrangements involving *NPM1* gene located at 5q35.1. Those include t(3;5)(q25.3;q35.1)/*NPM1::MLF1*, t(5;6)(q35.1;q23.1)/*NPM1::CCDC28A*, t(5;18)(q35.1;q21.2)/*NPM1::HAUS1*, and t(5;17)(q35.1;q21.1)/*NPM1::RARA*, which lead to the expression of NPM1 fusion proteins in AML [5–10]. These *NPM1* rearrangements are considerably rarer, and their pathogenic roles remain less well understood compared to those of NPM1c. However, a series of recent studies has begun to shed light on the diverse functions of NPM1 fusion proteins and the clinical features associated with *NPM1*-rearranged (NPM1-r) AML.

In this review, we summarize the clinical characteristics of NPM1-r AML and, incorporating our own data [11], provide an overview of current concepts regarding how NPM1 fusions contribute to leukemogenesis.

### Structure of NPM1 and NPM1 fusion genes

**Fig. 1:**
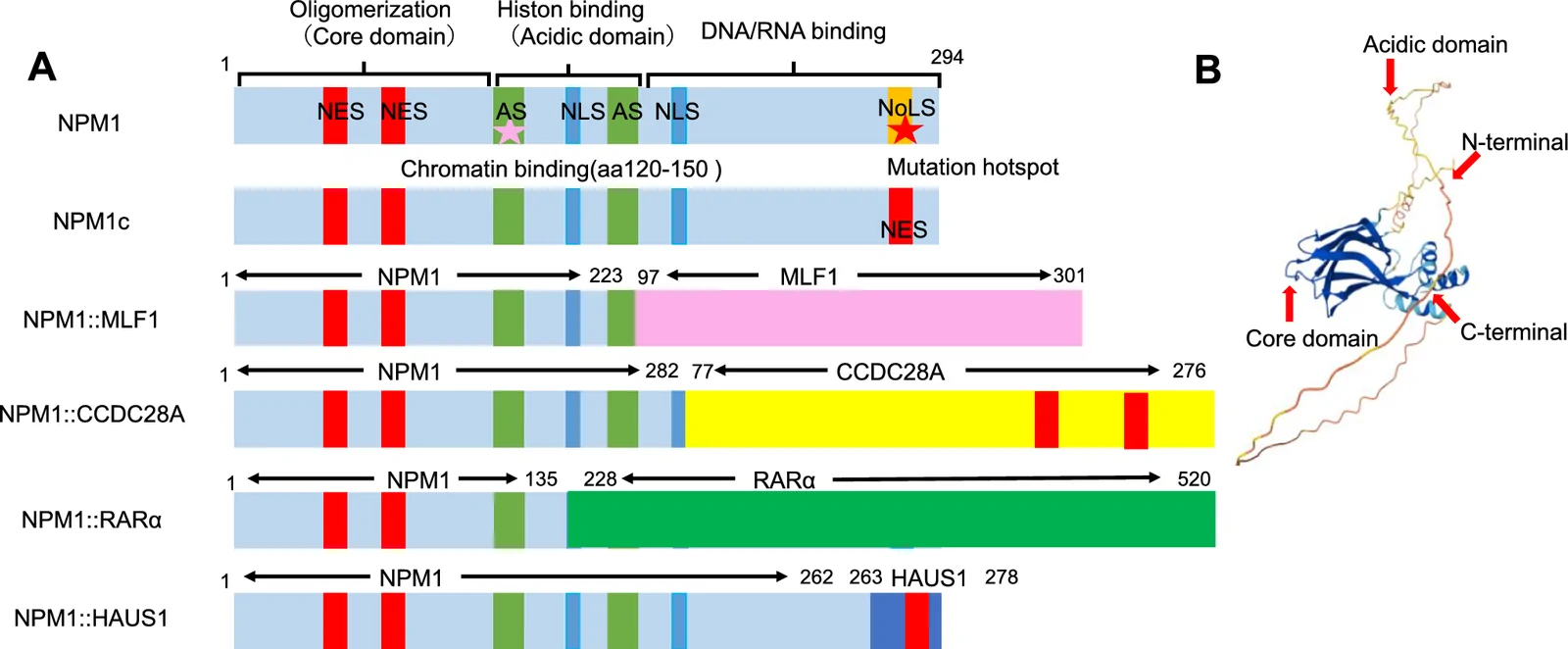
Structures of NPM1, NPM1c, and NPM1 fusion genes **A** Schematic representation of the primary structure of *NPM1* and its mutants. In all AML-associated *NPM1* mutants, the nucleolar localization signal (NoLS) is disrupted, leading to altered subcellular localization. NES, nuclear export signal; AS, acidic stretch; NLS, nuclear localization signal; NoLS, nucleolar localization signal. **B** Three-dimensional structure of NPM1

Human *NPM1* is located on chromosome 5q35 and comprises 12 exons. The protein consists of an N-terminal core domain, an acidic region, a nuclear localization signal (NLS), and a disordered C-terminal region (Fig. 1). The highly conserved N-terminal core domain mediates oligomerization and protein–protein interactions. The acidic region contains multiple acidic residues, forming a negatively charged stretch that contributes to histone binding. The C-terminal portion contains clusters of basic, positively charged residues followed by aromatic residues, which facilitate nucleic acid binding [12]. Wild-type NPM1 carries out a broad range of cellular functions, including nucleolar organization via liquid–liquid phase separation, regulation of ribosome biogenesis and export, DNA repair, centrosome duplication, and responses to nucleolar stress [13–15]. Nevertheless, the full spectrum of NPM1’s physiological roles has yet to be defined.

*NPM1* possesses multiple signals that regulate nucleocytoplasmic trafficking, functioning as a shuttling chaperone protein. The major signals include the nuclear export signal (NES), nuclear localization signal (NLS), and nucleolar localization signal (NoLS). The NES is recognized by the nuclear export receptor exportin 1 (XPO1), also known as chromosomal region maintenance 1 (CRM1), and facilitates the export of proteins from the nucleus to the cytoplasm. In contrast, the NLS is recognized by importin-α/β and mediates nuclear import from the cytoplasm. The NoLS directs proteins to the nucleolus and, in *NPM1*, is located within exon 12 [16]. Exon 12 is a mutation hotspot, where a typical four-base pair insertion results in a frameshift that leads to the loss of the NoLS and the acquisition of a novel C-terminal NES.

Importantly, a similar principle applies to NPM1 fusion proteins (Fig. 1). In fusions such as *NPM1::MLF1*, *NPM1::CCDC28A*, *NPM1::HAUS1*, and *NPM1::RARA*, the exon 12 region of *NPM1* is absent, resulting in the loss of the NoLS. Furthermore, fusion partners such as *CCDC28A* and *HAUS1* possess their own C-terminal NES motifs [7, 8]. When fused to *NPM1*, these NES motifs provide an additional NES, thereby producing a fusion protein that mimics NPM1c, allowing it to be exported to the cytoplasm.

### Clinical features of *NPM1*-rearranged AML

To elucidate the clinical features and outcomes of NPM1-r AML, we reviewed cases reported in the literature. Among the identified rearrangements, *NPM1::MLF1* is the most prevalent, accounting for approximately 0.2–0.5% of AML cases, including secondary AML following myelodysplastic syndromes [5]. A previous study detailed 69 cases of *NPM1::MLF1* AML [17], the key findings of which are summarized in Table 1. Table 2 summarizes the additional cases we incorporated into this study, comprising 8 cases of *NPM1::MLF1*, 3 of *NPM1::CCDC28A*, 2 of *NPM1::HAUS1*, and 9 of *NPM1::RARA* AML. This comprehensive analysis highlights critical observations regarding blast characteristics, co-occurring mutations, age distribution, and prognosis.

**Table 1:** Clinicopathological characteristics and outcomes of reported AML and MDS cases harboring *NPM1::MLF1* [17]

Category	Findings
Total reported AML and MDS cases with NPM1::MLF1	69
Average age	38.9 years
FAB classification (AML)	M2: 18 cases; M4: 11 cases; M6: 12 cases; M5: 2cases
AML with myelodysplasia-related changes (AML-MRC)	20
• MDS only	12
• MDS → AML	8
Cases with available sex information	51
Sex distribution	Male predominance (Male:Female = 34:17)
Cases with available outcome data	45
Deaths (among cases with outcome data)	20
Survival after HSCT	4/7

Of 69 reported AML and MDS cases harboring *NPM1::MLF1*, outcome and sex were documented in 45 and 51 patients, respectively. The cohort showed a male predominance (Male:Female = 34:17) and a mean age of 38.9 years. AML, acute myeloid leukemia; MDS, myelodysplastic syndrome; HSCT, hematopoietic stem cell transplantation

**Table 2:** Patient characteristics of NPM1-rearranged AML

	Age	Sex	WBC (μl)	PB Blast (%)	CD34 expression in Blast	FAB	Treatment	SCT	Outcome	OS (month)	Relapse	Other somatic mutation	Refs
NPM1-MLF1	12.9	M	14,400	35.6	N.A	Secondary AML from MDS	AML-05	Done	Alive	45	No	None (Target genes)	[6]
	3	F	80,400	30	Negative	M2	LAME 91	No	Dead	2	No	None (Target genes)	[5]
	45	F	4300	8	Negative	M2	ALFA-9802	Done	Alive	84	Yes	WT1 (Target genes)	[5]
	49	F	35,500	55	Negative	M2	ALFA-9802	No	Dead	18	Yes	FLT3-ITD (Target genes)	[5]
	34	F	24,900	85	Negative	M2	ALFA-0702	No	Dead	14	Yes	FLT3-ITD, IDH2 (Target genes)	[5]
	15	F	4300	9	Negative	M2	ELAM02	No	Alive	16	No	None (Target genes)	[5]
	2	M	28,100	4	Negative	M4	ELAM02	Done	Alive	14	No	None (Target genes)	[5]
	22	F	17,000	31	Negative	M2	ALFA-0702	Done	Dead	7	No	IDH2 (Target genes)	[5]
NPM1:: CCDC28A	1.4	F	16,600	4.6	N.A	M2	AML-05	Done	Dead (Infection)	6	No	FLT3-ITD (Target genes)	[6]
	68	M	32,000	N.A	Negative	N.A	IC	N.A	Loss FU	N.A	Yes	FLT3-ITD, KRAS DNMT3A (Whole exome)	[7]
	61	F	84,900	N.A	Negative	N.A	Actinomycin D	N.A	Dead (Relapse)	N.A	Yes	IDH2 (Whole exome)	[7]
NPM1::HAUS1	63	F	N.A	N.A	Negative	N.A	N.A	N.A	Dead (Infection)	1	No	FLT3-ITD, DNMT3A (Whole exome)	[8]
	78	M	N.A	N.A	Negative	N.A	N.A	N.A	Dead (Relapse)	18	Yes	FLT3-ITD (Whole exome)	[8]
NPM1::RARA	5	M	49,300	N.A	Negative	M3	ATRA + IC	No	Alive	N.A	No	N.A	[9]
	2.5	F	N.A	N.A	N.A	M3	ATRA + IC	Done	Dead	7	Yes	N.A	[10]
	12	M	N.A	N.A	N.A	M3	ATRA + IC	Done	Dead	8	Yes	N.A	[10]
	9	F	17,000	N.A	N.A	M3	ATRA + IC	No	Alive	29	No	N.A	[10]
	12	M	15,700	N.A	N.A	M3	ATRA	No	Dead (DIC)	5 days	No	N.A	[10]
	29	M	2900	N.A	N.A	M3	ATRA + IC	No	Dead	22	Yes	N.A	[10]
	64	M	15,000	0	Negative	M3	ATRA + IC	No	Alive	18	No	N.A	[10]
	4	M	25,560	0	N.A	M3	ATRA + IC	No	Alive	N.A	No	N.A	[10]
	51	M	4200	0	Negative	M3	ATRA + IC	No	Alive	2	No	N.A	[10]

M, male; F, female; WBC, white blood cell count; SCT, stem cell transplantation; OS, overall survival; FAB, French–American–British (FAB) classification; IC, standard intensive chemotherapy; FU, Follow-Up

*NPM1::RARA* was exclusively identified in acute promyelocytic leukemia (APL) treated with all-trans retinoic acid (ATRA)-based regimens [9, 10]. These findings suggest that *RARA* is the primary driver in this AML subtype, whereas the leukemogenic contribution of NPM1 remains undefined. Conversely, other NPM1-r AMLs exhibit phenotypic similarities to NPM1c-AML. Specifically, CD34 expression on blasts was consistently negative where data were available. Furthermore, co-occurring mutations such as *FLT3*-ITD, *DNMT3A, WT1,* and *IDH2*—common in NPM1c-AML—were frequently observed. The lack of CD34 expression and the mutational landscape closely mirror NPM1c-AML, implying shared leukemogenic mechanisms [18, 19].

Despite these similarities, NPM1-r AML presents distinct clinical features. Of the 22 cases analyzed (Table 2), 12 were pediatric or young adult patients, suggesting a predilection for younger individuals. Clinical outcomes indicate a poorer prognosis for NPM1-r AML compared to NPM1c-AML, characterized by high mortality (57.1%) and relapse (40.9%) rates. These results underscore the need for therapeutic strategies specifically tailored to NPM1-r AML. However, given the limited sample size, further large-scale studies are warranted to validate these trends.

### Molecular pathogenesis of NPM1-r AML

- (1) Leukemogenic activities of NPM1 fusion proteins

Although the functions of NPM1 fusion proteins have remained largely unknown, recent studies have gradually unveiled their leukemogenic activities. For instance, *NPM1::MLF1* alone is insufficient to enhance the colony-forming ability of normal mouse bone marrow cells. However, it can augment colony-forming activity in NPM1^±^ bone marrow progenitors, accompanied by increased expression of the Hoxa9, Hoxa10, and Meis1 [20]. Furthermore, *NPM1::MLF1*-transduced mouse bone marrow cells occasionally induce AML in a mouse retroviral gene transduction and transplantation model [11]. These findings suggest that *NPM1::MLF1* possesses weak leukemogenic activity, which requires additional cooperating events—such as reduced activity of wild-type *NPM1* and other mutations—for full leukemic transformation.

In contrast, *NPM1::CCDC28A* exhibits significantly stronger leukemogenic activity. *NPM1::CCDC28A*-transduced mouse bone marrow progenitors acquire extensive serial replating capacity in vitro and consistently induce AML in nearly all recipient mice [11]. Given that *CCDC28A* also forms the *NUP98::CCDC28A* fusion gene in T-cell leukemia [21], it is likely that the oncogenic activity of CCDC28A further enhances the leukemic potential of *NPM1::CCDC28A*.

*NPM1::RARA* has been shown to function comparably to *PML::RARA*, a well-established driver of APL. Ectopic expression of *NPM1::RARA* in mouse bone marrow cells induces APL-like phenotypes both in vitro and *in vivo*. Moreover, transgenic mice expressing *NPM1::RARA* develop an APL-like disease that responds to ATRA treatment similarly to *PML::RARA*-positive APL [22, 23]. Thus, *NPM1::RARA* appears to resemble *PML::RARA* more closely than other NPM1 fusion proteins.

- (2) Intracellular behavior of NPM1 fusion proteins

The formation of NPM1c or *NPM1* rearrangements invariably results in the loss of the nucleolar localization signal (NoLS) and, in many cases, the acquisition of a novel C-terminal nuclear export signal (NES) (Fig. 1). The newly generated C-terminal NES is preferentially recognized by XPO1 over the N-terminal NES, promoting the cytoplasmic localization of mutant NPM1 [24]. The NPM1 fusion proteins—NPM1::MLF1, NPM1::CCDC28A, and NPM1::HAUS1—all lack the NoLS and have been reported to localize to the cytoplasm [7, 8]. Because *CCDC28A* and *HAUS1* carry NESs, *NPM1::CCDC28A* and *NPM1::HAUS1* acquire an additional NES, similar to NPM1c. By contrast, *MLF1* does not contain a C-terminal NES; therefore, *NPM1::MLF1* does not gain a new NES. Our findings show that *NPM1::CCDC28A* exhibited more prominent cytoplasmic localization than *NPM1::MLF1* [11], consistent with the notion that the acquisition of a new NES enhances cytoplasmic trafficking. Moreover, given that *NPM1::CCDC28A* demonstrates stronger leukemogenic activity than *NPM1::MLF1*[11], this observation suggests that the degree of cytoplasmic mislocalization may be linked to leukemogenic potential. Thus, the aberrant redistribution of NPM1—normally restricted to the nucleolus—to the cytoplasm is a unifying feature of AML cells with both NPM1c and *NPM1* rearrangements.

Wild-type NPM1 is essential for cell survival, and its complete loss is embryonically lethal. Consequently, *NPM1* mutations and rearrangements are always heterozygous. NPM1c is known to form heterodimers with the wild-type protein, escorting it to the cytoplasm and significantly impairing nuclear NPM1 function. In addition, NPM1c aberrantly sequesters nuclear proteins, such as ARF, HEXIM1, FBW7, MIZ1, CTCF, PU.1, and caspases-6/8, to the cytoplasm [14, 25, 26]. As a result, both the nuclear function of NPM1 itself and the nuclear regulatory networks associated with NPM1 are disrupted in NPM1c-AML, likely contributing to leukemogenesis. A similar disruption of NPM1 function and its associated nuclear proteins is likely to occur in NPM1-r AML, although this hypothesis requires experimental confirmation in future studies.

- (3) Chromatin regulation by NPM1 fusion proteins

A distinctive feature of AML with *NPM1* abnormalities is the sustained high-level expression of *HOX* genes. Under physiological conditions, *HOX* genes such as *HOXA9* are highly expressed in hematopoietic stem cells but are downregulated during differentiation. However, in *NPM1*-mutated AML, *HOX* expression remains at levels comparable to those in normal stem cells, driving leukemic transformation. Genome-editing studies have demonstrated that the upregulation of *HOX* genes is strictly dependent on the presence of NPM1c [27]. Pharmacologic inhibition of XPO1, which blocks the export of NPM1c, reduces *HOX* expression and promotes differentiation of *NPM1*-mutated AML cells [28]. These findings establish a direct causal relationship between *NPM1* mutation and *HOX* activation.

Recent studies have proposed that a small fraction of mutant *NPM1* retained in the nucleus directly binds to the promoter regions of *HOX* clusters and *MEIS1*, thereby regulating transcription [29, 30]. XPO1, traditionally recognized as a nuclear export factor, has been identified as a central mediator of this process. XPO1 can bind directly to chromatin, and chromatin association of mutant *NPM1* requires XPO1. It has been proposed that XPO1 first associates with chromatin and subsequently recruits NPM1c. NPM1c, via its acidic domain (amino acids 120–150), forms a complex with KMT2A/MLL1 and RNA polymerase II on chromatin to activate target-gene transcription. More recent work has shown that NPM1c phase-separates to form nuclear condensates, termed the "coordinating body" (C-body), which recruits XPO1, NUP98, KMT2A, and MENIN. The C-body plays a crucial role in regulating gene expression and promoting leukemic proliferation [31]. Importantly, XPO1 inhibition disrupts the association of mutant *NPM1* with chromatin, leading to downregulation of *HOX* and *MEIS1* expression. This highlights the XPO1-mediated pathway as a promising therapeutic target.

Interestingly, NPM1 fusion proteins appear to function similarly to NPM1c. Both *NPM1::MLF1* and *NPM1::CCDC28A* upregulate *HOX* genes. NPM1::CCDC28A binds to HOX promoters and activates their transcription, and this effect is abolished by XPO1 inhibition [11]. These findings suggest that NPM1 fusion proteins, like NPM1c, cooperate with XPO1 to sustain abnormal *HOX* expression and drive leukemogenesis.

**Fig. 2:**
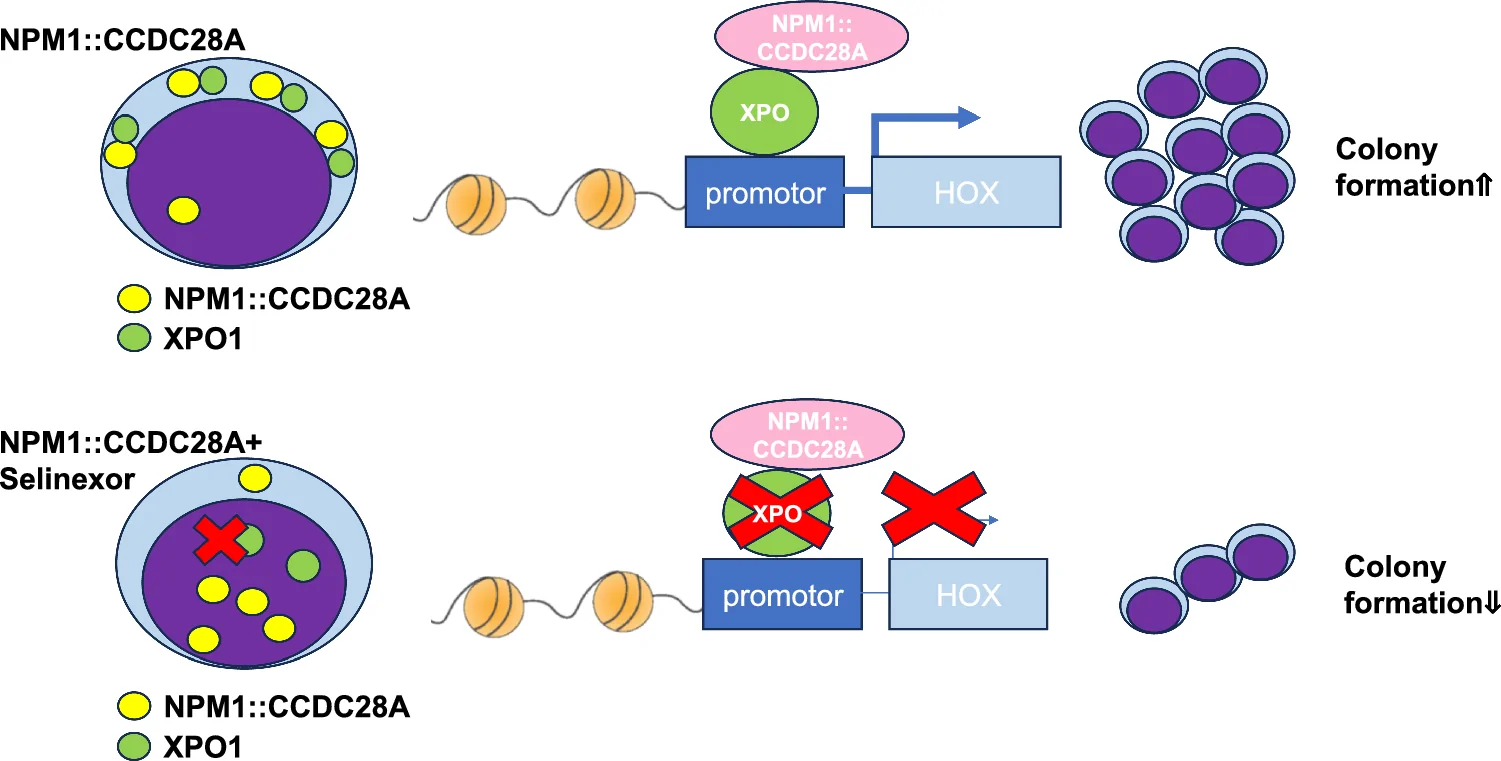
NPM1:CCDC28A predominantly localizes in the cytoplasm and induces HOX gene expression through XPO1. NPM1::CCDC28A binds to XPO1 and predominantly localizes in the cytoplasm. A portion of NPM1:CCDC28A in the nucleus is recruited to the promoters of HOX gene clusters by XPO1, where it promotes HOX gene transcription. When XPO1 is inhibited by selinexor, NPM1::CCDC28A dissociates from chromatin, resulting in HOX gene downregulation and reduced colony formation

In conclusion, sustained *HOX* expression in *NPM1*-mutated AML is likely dependent on the interaction between mutant NPM1 and XPO1. XPO1 is now recognized to have dual roles: mediating the nuclear export of mutant NPM1 and anchoring them to chromatin (Fig. 2). It remains to be determined which of these functions is more critical for leukemogenesis. The prevailing model suggests that NPM1c and NPM1 fusion proteins bind to HOX gene promoters in cooperation with XPO1, maintaining constitutive *HOX* expression and driving leukemia.

### Therapeutic strategies for NPM1-r AML

- (1) XPO1 inhibitors

XPO1 is involved in both nuclear export and chromatin recruitment of oncogenic complexes, making it an important therapeutic target in AML with NPM1c and *NPM1* rearrangements. In a mouse leukemia model harboring NPM1c and *FLT3*-ITD, treatment with an XPO1 inhibitor significantly prolonged survival [28]. Selinexor is a selective inhibitor of nuclear export that specifically targets XPO1/CRM1, a critical mediator of nuclear protein export, and chromatin recruitment of oncogenic complexes. In our study, XPO1 inhibition suppressed colony-forming capacity, inhibited *HOX* gene expression, and restored the nuclear localization of *NPM1::CCDC28A* in mouse AML cells transformed by *NPM1::CCDC28A* [11]. Clinical trials of selinexor in AML are currently ongoing, with its safety already confirmed in a phase I trial for relapsed/refractory AML. Beyond its activity as a single agent, selinexor is expected to exert synergistic effects when combined with conventional chemotherapy [32].

The second-generation XPO1 inhibitor, eltanexor, offers advantages over selinexor, including lower toxicity and limited penetration of the blood–brain barrier. Eltanexor provides more sustained XPO1 inhibition and has demonstrated efficacy in patient-derived xenograft models of NPM1c-AML [33]. However, it is important to note that XPO1 inhibitors are not specific to NPM1; they also affect the nuclear export of other proteins, such as TP53 and CDKN1A [16]. Therefore, careful interpretation is required to assess the extent to which their antileukemic effects are directly attributable to *NPM1*.

- (2) Menin inhibitors

Menin inhibitors are emerging as promising agents for the treatment of *NPM1*-mutated AML. Menin functions as a cofactor of *MLL1 (KMT2A)*, promoting H3K4 methylation and transcriptional activation of target genes such as *HOXA9* and *MEIS1*. Although these agents were initially developed to target leukemia with *MLL1* rearrangements, Menin inhibition is also expected to be effective against NPM1c-AML due to the cooperative role of NPM1c with *MLL1* in enhancing HOX/MEIS transcription.

Indeed, novel Menin inhibitors, such as MI-3454 and VTP-50469, have demonstrated potent antileukemic activity in murine models of NPM1c-AML [34]. The oral Menin inhibitor revumenib (SNDX-5613) selectively disrupts the Menin–MLL interaction and induced complete remission (CR) or CR with partial hematologic recovery in approximately 30% of relapsed AML patients, with an acceptable safety profile in a first-in-human phase I trial (NCT04065399) [35]. In our study, AML cells expressing *NPM1::CCDC28A* were highly sensitive to Menin inhibition [11], suggesting that Menin inhibitors may be effective not only in NPM1c-AML but also in NPM1-r AML.

Furthermore, the combined use of Menin and XPO1 inhibitors has shown synergistic effects in murine models of NPM1c-AML [36]. Similarly, the combination therapy outperformed monotherapy in human cord blood cells and murine bone marrow cells expressing *NPM1::CCDC28A* [11]. These findings highlight the potential of combination therapy with XPO1 and Menin inhibitors for NPM1-r AML, warranting further investigation in future clinical trials.

## Conclusions and future perspectives

The leukemogenic mechanisms of NPM1 fusion proteins, excluding NPM1::RARA, are increasingly recognized to closely resemble those of NPM1c. In particular, key features such as the cytoplasmic localization of NPM1 fusion proteins, their interaction with XPO1, involvement in chromatin-level regulation, and induction of aberrant *HOX* gene expression—all of which contribute to leukemogenesis—are shared by both NPM1c and NPM1-r AML. This molecular similarity suggests that therapeutic approaches effective for NPM1c-AML, such as XPO1 inhibitors and Menin inhibitors, may also be beneficial for NPM1-r AML.

At the same time, differences among fusion partners must be carefully considered. The biological behavior and therapeutic vulnerabilities of each *NPM1* fusion appear to vary. For instance, *NPM1::RARA* exhibits functions similar to other *RARA*-rearranged genes, such as *PML::RARA*, and should therefore be considered separately from other *NPM1* rearrangements. *NPM1::CCDC28A* appears to have stronger leukemogenic potential than other *NPM1* fusions, which may necessitate more intensive treatment strategies, such as stem cell transplantation. The generally poorer prognosis associated with NPM1-r AML likely reflects the additive oncogenic activities of these specific fusion partners, which amplify the core leukemogenic drive of the *NPM1* fusion and culminate in a more aggressive clinical phenotype.

Several critical questions remain unresolved. Why does XPO1 possess two seemingly opposing functions—nuclear export and chromatin binding—and which of these functions is more critical in NPM1c and NPM1-r AML? Additionally, why do only mutant or fusion NPM1 proteins gain access to chromatin? Elucidating the protein interaction networks of wild-type NPM1, mutant NPM1, and NPM1 fusion proteins in both the nucleus and cytoplasm may uncover novel signaling pathways and potential therapeutic targets. Continued advances in both basic and clinical research are essential, and hold promise for improving outcomes in NPM1-r AML.

## Data Availability

No new data were generated or analyzed in this study. Data sharing is not applicable to this article.
